# Hyperglycemic hemifacial spasm: A case report

**DOI:** 10.1111/cns.13739

**Published:** 2021-10-04

**Authors:** Yanxing Chen, Luhang Jin, Yuyu Xu, Zheyu Li, Baorong Zhang, Feng Gao

**Affiliations:** ^1^ Department of Neurology The Second Affiliated Hospital School of Medicine Zhejiang University Hangzhou China

**Keywords:** blepharospasm, differential diagnosis, hemifacial spasm, hyperglycemic, MRI

## 1 INTRODUCTION

Hemifacial spasm (HFS) is a movement disorder characterized by either brief or persistent, intermittent twitching of the muscles innervated by the facial nerve, commonly resulting from compression of the seventh cranial nerve and also can be secondary to abnormal metabolic conditions.^1^ The most commonly reported and best‐recognized movement disorder secondary to hyperglycemia is hemichorea.^2^ In this study, we report an unusual case presenting with hemifacial spasm as the initial manifestation of uncontrolled hyperglycemia, which resolved rapidly following the correction of hyperglycemia. This case broadens our understanding of movement disorders associated with hyperglycemia.

This study followed the tenets of the Declaration of Helsinki, and was performed according to the guidelines of the Second Affiliated Hospital of Zhejiang University School of Medicine. Written informed consents were obtained from the patient.

## 2 CASE REPORT

**FIGURE 1:**
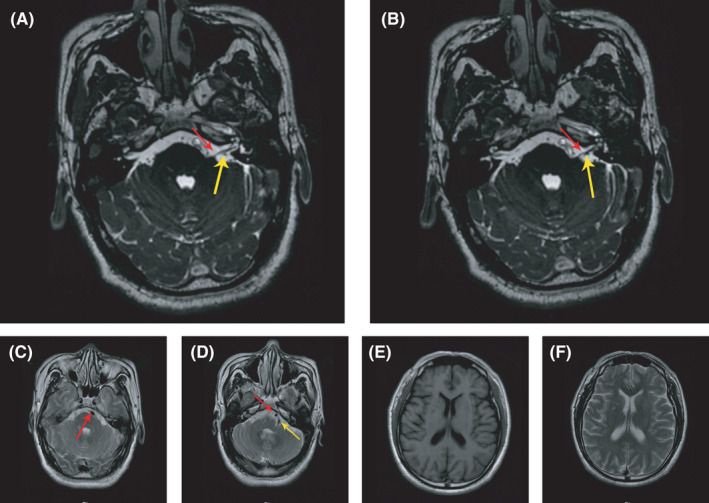
(A and B) Facial nerve MRI shows branching vessels adjacent to the left facial nerve. The red arrow shows the left facial nerve, and the yellow arrow shows the branching vessel. (C and D) Magnetic resonance angiography (MRA) shows tortuous expansion of vertebrobasilar artery. The red arrow shows the tortuous dilated basilar artery, and the yellow arrow shows the tortuous left vertebral artery. (E and F) T1‐weighted and T2‐weighted brain MRI show no obvious hyperintensity of the basal ganglia

A 46‐year‐old male patient was referred to our neurology department due to a 9‐day history of involuntary contractions of his left facial expression muscles, involving both the upper and lower face (Video S1). Each episode lasted less than a minute and occurred multiple times per day, particularly during speaking and eating. The patient was alert during episodes. The symptom disappeared during sleep, and no other conditions such as involuntary movement of the limbs, dizziness, slurred speech, limb weakness, and double vision were present. He had been medicated with baclofen, vitamin B12, vitamin B1, and oxcarbazepine on the fourth day after the symptom onset in another hospital, but with no relief. His prior medical history was unremarkable. On neurological examination, he was fully alert and oriented, and no other signs were observed except for episodes of hemifacial spasm. CT scan of the brain and Electroencephalogram prior to admission were normal. His blood glucose level on admission was 26.4 mmol/L, and the glycosylated hemoglobin A1C (HbA1C) level was 13.3%, with normal arterial blood gas (ABG) analysis and negative urine ketones. Lumbar puncture showed high CSF glucose level of 6.55 mmol/L with normal chloride and protein levels and cell counts. Brain magnetic resonance imaging (MRI) revealed vertebrobasilar dolichoectasia (VBDE) (Figure 1C,D) with branching vessels adjacent to the left facial nerve (Figure 1A,B). No obvious hyperintensity of the basal ganglia was observed on T1‐weighted or T2‐weighted images (Figure 1E,F).

After admission, the patient's blood glucose levels were controlled with insulin (Table 1). Oral hypoglycemic drugs including metformin, acarbose, and gliclazide were also prescribed during the following days. A rapid improvement of the patient's symptom was observed after blood glucose normalization on the second hospital day. No additional episodes of hemifacial spasm occurred since the third day of hospitalization when a steady correction of the hyperglycemia was achieved (Video S2). The patient was discharged home with oral hypoglycemic drugs. During the 2‐year follow‐up, he had good adherence to the medications and denied new episodes of involuntary movements. Repeated facial nerve MRI did not show any obvious changes as compared to the former one (Figure S1).

**TABLE 1:** Blood glucose levels

Date	Blood glucose levels (mmol/L)							
	6 a.m.	8 a.m.	11 a.m.	13 p.m.	17 p.m.	19 p.m.	21 p.m.	Random blood glucose
Day 1					20.7	9.3	13.8	26.4 (14:30 p.m.)
Day 2	14.9	26.4	19.5	/	9.5	/	11.3	
Day 3	10.5	11.9	11.4	/	7.7	/	9.4	
Day 4	10.9	10.7	12.3	/	/	8.2	6.1	

## 3 DISCUSSION

Movement disorders mainly hemichorea is a rare complication of poorly controlled diabetes mellitus (DM) in elderly patients, which is mostly reported in the Asian population. Brain MRI may reveal hyperintensity of caudate and lenticular nucleus on T1‐weighted MR images without surrounding edema or mass effect.^2^, ^3^ Our patient presented with acute onset of hemifacial spasm with uncontrolled DM, which completely resolved after controlling the plasma glucose levels. The most common cause for primary hemifacial spasm is the local demyelination of the facial nerve resulting from the compression by an ecstatic or aberrant blood vessel.^4^ Causes for secondary hemifacial spasm, including arteriovenous malformation, brainstem lesions, infection, structural abnormalities of the posterior cranial fossa, parotid tumors, and Bell's palsy, were unlikely due to the normal brain MR imaging and negative medical history of the patient. Focal cortical seizure involving the facial muscles should also be ruled out. However, repetitive electroencephalogram recording during the episodes were also unremarkable. The complete resolution of the hemifacial spasm following the correction of hyperglycemia indicates that hemifacial spasm, like hemichorea, might be the complication of uncontrolled DM. This is a rare manifestation of movement disorders associated with DM. Only one report so far showed hemifacial spasm as the predominant clinical manifestation in two patients with diabetic ketoacidosis, which subsided with conventional therapy for diabetic ketoacidosis.^5^ In the current case, urine analysis revealed negative for ketones and the ABG was normal, indicating a nonketotic hyperglycemic state. The underlying pathophysiological mechanisms are unknown. For this case, we believe that both VBDE and the hyperglycemic state might be involved. VBDE has been reported to be associated with increased prevalence of hemifacial spasm, which may be related with increased risk of vascular compression of the facial nerve resulting from vascular crowding of the posterior fossa space.^6^ Transient ischemia induced by hyperglycemic hyperosmolarity may cause the facial nerve irritable to the compression of the adjacent branching vessels, resulting in the involuntary contraction of the face.^7^

In the current study, we report a case presenting with hemifacial spasm as the initial manifestation of diabetes, which completely resolved after glycemic control. To the best of our knowledge, we are the first to report a case of hemifacial spasm associated with nonketotic hyperglycemia, indicating the necessity to consider hyperglycemia as a differential diagnosis for hemifacial spasm. The underlying pathophysiological mechanisms are elusive, which need further investigation.

## CONFLICT OF INTERESTS

Dr. Chen, Luhang Jin, Yuyu Xu, Zheyu Li, Dr. Zhang and Dr. Gao reports no disclosures.

## AUTHOR CONTRIBUTIONS

Yanxing Chen, Luhang Jin: Analyzed and interpreted the data and wrote the manuscript. Yuyu Xu and Zheyu Li: Analyzed and interpreted the data. Baorong Zhang: Designed and interpreted the data. Feng Gao: Designed and interpreted the data, revised the manuscript.

## Supporting information

Figure S1

Video S1

Video S2

Supplementary Material

## Data Availability

The data that support the findings of this study are available from the corresponding author upon reasonable request.
