# Supplementary figures and images for: Hyperglycemic hemifacial spasm: A case report
Source: CNS Neurosci Ther. 2021 Oct 4;27(12):1614–6. doi: 10.1111/cns.13739 (PMC8611785; doi:10.1111/cns.13739)

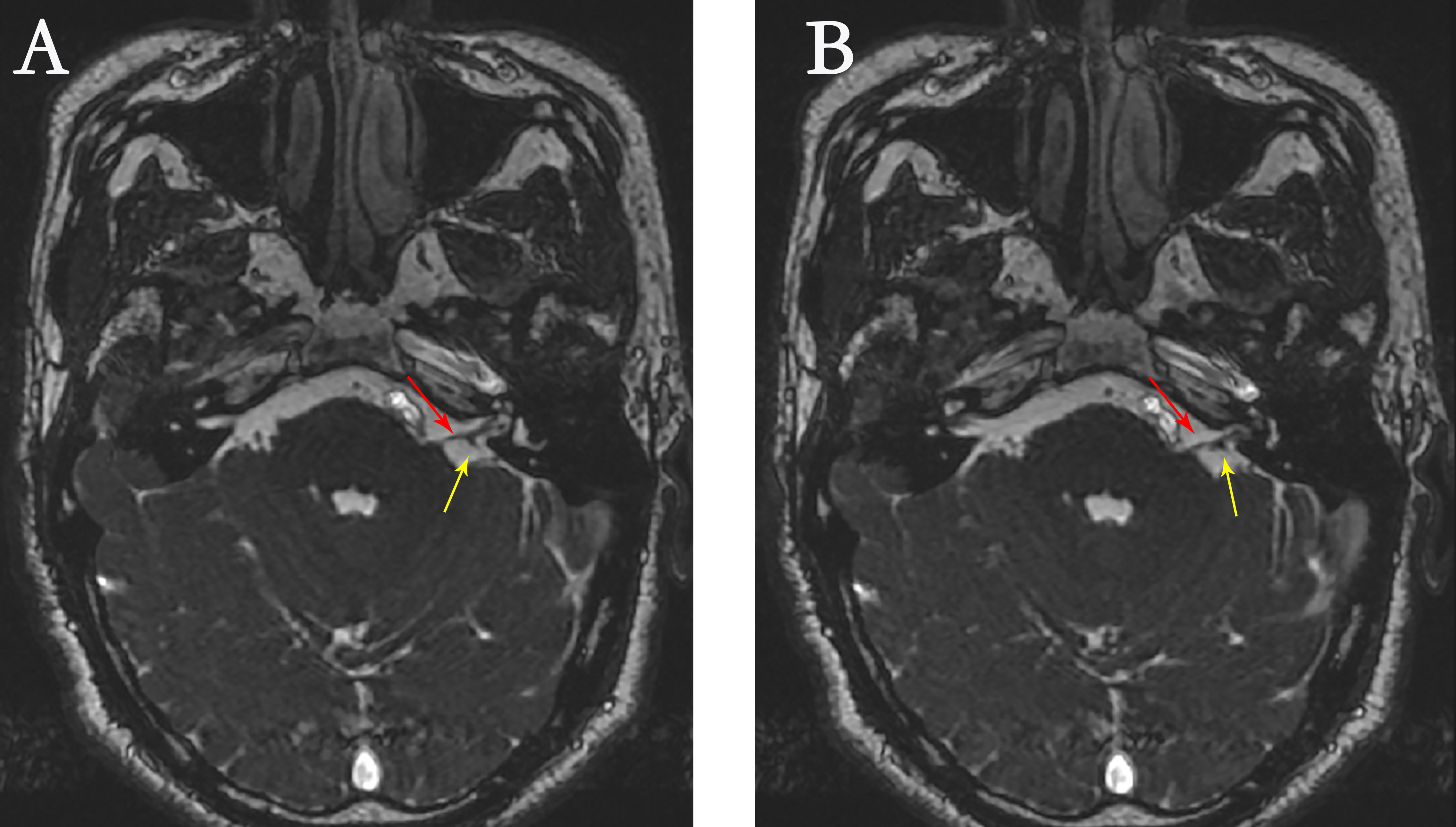

Supplement: Supplementary file 1 — Figure S1 [file CNS-27-1614-s001.tif]
