# Supplementary material for: Hyperglycemic hemifacial spasm: A case report
Source: CNS Neurosci Ther. 2021 Oct 4;27(12):1614–6. doi: 10.1111/cns.13739 (PMC8611785; doi:10.1111/cns.13739)
Supplement: Supplementary file 4 — Supplementary Material [file CNS-27-1614-s003.docx]

**Figure legends**

Supplementary Figure S1. (A and B) Facial nerve MRI shows branching vessels adjacent to the left facial nerve. The red arrow shows the left facial nerve, and the yellow arrow shows the branching vessel.
